# Sociodemographic factors associated with the first administration of anti-seizure medication in patients with focal epilepsy in Western China

**DOI:** 10.1186/s12883-021-02282-w

**Published:** 2021-06-29

**Authors:** Qiong Zhu, Yi Guo, Shuai Ma, Lili Yang, Zhonghua Lin, Hongbin Sun, Guangzong Li, Liang Yu

**Affiliations:** 1 Department of Neurology, Sichuan Provincial People’s Hospital, University of Electronic Science and Technology of China, Chengdu, 610072 China; 2 Chinese Academy of Sciences Sichuan Translational Medicine Research Hospital, Chengdu, 610072 China; 3 Department of Neurology, The Sixth People’s Hospital of Chengdu, Chengdu, 610051 China; 4 Sichuan Provincial Center for Mental Health, The Center of Psychosomatic Medicine of Sichuan Provincial People’s Hospital, University of Electronic Science and Technology of China, Chengdu, 611731 China

**Keywords:** Initial treatment, Anti-seizure medications, Sociodemographic factors, Focal epilepsy, Western China

## Abstract

**Background:**

Epilepsy is a severe chronic neurologic disease with a prevalence of 0.7% worldwide; anti-seizure medications (ASMs) are the mainstay of epilepsy treatment. The effects of sociodemographic factors on the characteristics of initial treatment in patients with newly diagnosed focal epilepsy in Western China are unknown. This study was conducted to explore sociodemographic factors associated with initial treatment characteristics.

**Methods:**

Patients with focal epilepsy on continuous ASM treatment who visited to our epilepsy center at Sichuan Provincial People’s Hospital between January 2018 and December 2019 were recruited. Data on initial treatment status and sociodemographic variables were obtained from the patients with a questionnaire designed by our researchers. We examined whether sociodemographic factors were associated with epileptic patients’ access to neurologists and prescriptions of individual ASMs**.**

**Results:**

A total of 569 patients completed this study. We found that patients with a higher education level, aged < 16 years, and with a higher household disposable income were more likely to receive treatment from a neurologist than their counterparts. Patients with a lower personal income level and who were treated at a junior hospital were more likely to receive prescriptions for carbamazepine, and those who were younger than 16 years were less likely to receive prescriptions for carbamazepine and oxcarbazepine. Patients with a higher education level, with a higher household disposable income level, who were younger than 16 years, and who were treated at a senior hospital were more likely to receive prescriptions for levetiracetam than their counterparts. Adult, female patients with focal epilepsy treated at a senior hospital were more likely to receive prescriptions for lamotrigine.

**Conclusions:**

This observation suggests that sociodemographic characteristics are associated with access to neurologists and prescriptions of individual antiepileptic drugs. These data may help public health officials establish guidelines for doctors and distribute resources according to the needs of different patient groups.

## Background

Epilepsy is a severe chronic neurologic disease, with a prevalence of 0.7% worldwide [1]. In mainland China, the prevalence of epilepsy in the periods of 2001–2005 and 2006–2010 were 6.76 and 6.62%, respectively [2]. Epilepsy may lead to physical injuries, poor quality of life, loss of health and sudden unexpected death [3, 4]. Anti-seizure medications (ASMs) are the mainstay of epilepsy treatment, and the number of available ASMs has increased steadily since 1989 [5]. Patients with newly diagnosed epilepsy have an approximately 50% chance of seizure control after the first monotherapy with an appropriate ASM [6]. Previous studies had revealed that epilepsy patients benefit from a seizure-free period and fewer ASM-relevant adverse events compared with those who frequently change their therapy [7, 8]. Therefore, the initial therapy for epileptic patients is important for long-term prognosis. Thus, in 2006 and 2013, the International League Against Epilepsy (ILAE) focused on the initial monotherapy for epilepsy and related syndromes and disclosed their evidence-based analysis for clinical practice [9, 10].

In China, it has been reported that only 67.8% of patients with newly diagnosed epilepsy receive rational antiepileptic initial treatment, 77.5% of which were monotherapies [11]. According to recent investigations, the epilepsy treatment gap varies significantly between different areas and ethnicities and is especially high in western China [12, 13]; this may due to the sociodemographic factors. The population of western China accounts for nearly one-third of China’s total population, and the population in western China is less educated than populations in other regions. Moreover, Western China has a lower development level than other regions in China in terms of the economy and medical care. The effects of sociodemographic factors on the characteristics of initial treatment in patients with newly diagnosed focal epilepsy in western China have never been discussed until now.

In this study, we aimed to investigate whether access to a neurologist and individual ASMs were associated with sociodemographic factors in newly diagnosed focal epilepsy patients. Our study showed that patients with a higher education level, younger age and higher household disposable income were more likely to receive treatment from a neurologist than their counterparts. The prescription of ASMs differed according to age, sex, education level, and household disposable income.

## Methods

### Study design and procedures

Between January 2018 and December 2019, all patients with focal epilepsy who came to our epilepsy center at Sichuan Provincial People’s Hospital, a tertiary referral epilepsy center in Western China, were invited to participate in our study. Patients were included if they met the following inclusion criteria: patients were required to have come from western China and to have received their initial treatment at a hospital in western China. They were also required to have a validated diagnosis of focal epilepsy and to have received ASM therapy. Patients were excluded if they or their caregivers could not recall information about the initial therapy. This study was approved by the ethics committee of Sichuan Provincial People’s Hospital. Our patients were all informed of the purpose of the study, and we obtained their written informed consent (the informed consent for patients under 16 years old was signed by their legal guardian).

### Sociodemographic status and initial ASM prescription

An ASM user was defined as having access to a neurologist if a neurologist dispensed any drug. The neurologist specialized in epilepsy and was registered in the National Health and Family Planning Commission of the People’s Republic of China (http://zgcx.nhfpc.gov.cn/doctorsearch.aspx).

The patients were divided into three groups according to their ages: a) juveniles were defined as people younger than 16 years old; b) adults were defined as people from 16 to 60 years old; and c) the elderly were people older than 60 years old. The patients were divided into three groups according to their education level (the education level of juveniles was substituted with the education level of their parents who came with them during their first visit to the hospital): primary school (less than 6 years of schooling), high school (7–12 years), and Bachelor’s degree and above (more than 12 years). The patients were divided into two groups according to their place of residence: a) living in city, i.e., places with more than 100,000 residents within 10 km of a municipal center and b) living in the countryside, i.e., far from a municipal center. The ASM users were classified into 2 layers according to their household disposable income, i.e., those with a disposable income over or under the average income of western China.

The hospitals where the epilepsy patients received initial treatment were divided into 3 levels: a) junior-grade hospitals, defined as those that provided basic medical services for residents; b) middle-grade hospitals, defined as those that provided medical services to a larger population in a prefecture in Western China; and c) senior-grade hospitals, also called tertiary referral hospitals, defined as hospitals that were equipped with video-EEG/magnetic resonance and other equipment, ensuring that patients with suspected epilepsy received a comprehensive examination by a neurologist who specialized in epilepsy for more than 15 years. Each hospital had at least one neurologist specialized in epilepsy.

### Statistical analysis

All analyses were performed with SPSS 22.0 software. Logistic regression was used to calculate the odds ratios (ORs) with 95% confidence intervals (CIs) as estimates of whether access to prescriptions by neurologists differed between subgroups of epileptic patients. Age, gender, educational level, residence and income level were entered together into this model. In addition to the crude estimates, the ORs were adjusted for all of the other variables in a separate model. We also compared the prescription patterns of the 5 most prescribed ASMs within each variable, which were adjusted by ORs with 95% CIs. Adjustments included differences in age, gender, educational level, residence, household disposable income level and hospital level.

## Results

### General characteristic of the study population

A total of 609 patients who visited our epilepsy center from January 2018 to December 2019 met the inclusion criteria, and 589 patients agreed to participate in the study (the response rate was 96.7%). A total of 569 patients completed the study. The sociodemographic characteristics were shown in Table 1. The mean age of the patients was 31.8 ± 17.3 years, and 54.3% were male. In total, 338 patients were treated with ASMs by neurologists.

**Table 1:** Characteristics of the study population

	Patients	%
Age	569	100
< 16Y	97	17
16-60Y	423	74.4
≥ 60Y	49	8.6
Sex		
Male	309	54.3
Female	260	45.7
Education level		
Primary school	132	23.2
High school	350	61.5
Bachelor’s degree and above	87	15.3
Residence		
Urban	286	50.3
Rural	283	49.7
Household income (Yuan/year)		
< 60,000	238	41.8
≥ 60,000	331	58.2
Prescribing doctor		
Neurologist	338	59.4
Non-neurologist	231	40.6
Hospital level		
Junior	454	79.8
Middle	53	9.3
Senior	62	10.9
Etiology		
Metabolic	67	11.8
Genetic	78	13.7
Immune	62	10.9
Structural	154	27.1
Infectious	45	7.9
Unknown	163	28.6
Seizure type		
Focal aware seizure	214	37.6
Focal impaired awareness seizure	355	62.4

### Sociodemographic differences in access to prescriptions by a neurologist

As shown in Table 2, patients with a higher education level and higher income level were more likely to be treated by a neurologist. Compared with elderly patients, patients aged <16 years were more likely to receive treatment from a neurologist.

**Table 2:** Percentages in those newly diagnosed with focal epilepsy and odds ratios (ORs) for treatment by a neurologist (*n* = 569)

	n (%)^a^	Crude OR (95% CI)	Adjusted OR^b^ (95% CI)
Age			
<16Y	(74) 76.3	2.46 (1.18–5.27)	2.41 (1.11–5.23)
16-60Y	(235) 55.6	0.85 (0.46–1.54)	0.69 (0.37–1.31)
≥ 60Y	(29) 59.2	1	1
Sex			
Male	(183) 59.2	0.94 (0.70–1.38)	0.98 (0.69–1.39)
Female	(155) 59.6	1	1
Education level			
Primary school	(80) 60.6	0.59 (0.33–1.05)	0.39 (0.19–0.77)
High school	(195) 55.7	0.48 (0.29–0.80)	0.48 (0.28–0.82)
Bachelor’s degree and above	(63) 72.4	1	1
Residence			
Urban	(186) 65.0	1.603 (1.14–2.25)	1.34 (0.93–1.94)
Rural	(152) 53.7	1	1
Household income (Yuan/year)			
≥ 60,000	(178) 74.8	1.68 (1.20–2.36)	1.56 (1.09–2.23)
< 60,000	(160) 48.3	1	1

*Y* Years, *CI* Confidence interval, *OR* Odds ratio

^a^ Percentage of patients treated by a neurologist

^b^ Separate odds ratios adjusted for all of the variables in the model

### Sociodemographic differences in access to individual ASMs

Table 3 shows the adjusted ORs of risk factors for receiving a prescription for individual ASMs. We found the following results: A) Patients with a lower personal income who were treated at a junior hospital were more likely to receive prescriptions of carbamazepine. B) Patients with a higher education level, a higher income level, and who were treated at a senior hospital were more likely to receive prescriptions of levetiracetam. In addition, patients younger than 16 years were more likely to receive prescriptions of levetiracetam. C) Women treated at a senior hospital were more likely to receive prescriptions of lamotrigine. In addition, patients age 16–60 years were more like to receive prescriptions of lamotrigine. D) Compared with elderly patients, those who were younger than 16 years were less likely to receive prescriptions of carbamazepine and oxcarbazepine. E) Patients with a lower personal income were more likely to receive a prescription for valproate, while patients who were treated at a junior hospital were less likely to receive valproate.

**Table 3:** Risk factors for receiving ASM therapy among epileptic patients on continuous ASM treatment (*n* = 569 ^a^)

	Valproate OR (95% CI)	Levetiracetam OR (95% CI)	Lamotrigine OR (95% CI)	Carbamazepine OR (95% CI)	Oxcarbazepine OR (95% CI)
Age					
<16Y	0.72 (0.32–1.61)	7.5 (2.61–21.77)	3.60 (0.73–18.09)	0.16 (0.03–0.90)	0.17 (0.06–0.46)
16-60Y	0.92 (0.46–1.87)	2.11 (0.77–5.78)	5.21 (1.18–22.96)	0.84 (0.30–2.35)	0.29 (0.13–0.65)
≥ 60Y	1	1	1	1	1
Gender					
male	1.31 (0.88–1.93)	0.79 (0.51–1.23)	0.45 (0.27–0.75)	0.98 (0.57–1.69)	0.87 (0.49–1.56)
female	1	1	1	1	1
Education level					
Primary school	2.16 (0.97–4.79)	0.41 (0.18–0.93)	0.49 (0.18–1.32)	0.61 (0.21–1.77)	2.51 (0.81–7.79)
High school	2.32 (1.22–4.39)	0.43 (0.23–0.79)	0.59 (0.31–1.16)	1.03 (0.49–2.18)	1.24 (0.47–3.24)
Bachelor’s degree and above	1	1	1	1	1
Residence					
Urban	1.06 (0.71–1.59)	0.72 (0.45–1.15)	1.11 (0.64–1.94)	1.13 (0.63–1.99)	0.80 (0.43–1.49)
Rural	1	1	1	1	1
Household income (Yuan/year)					
< 60,000	1.93 (1.27–2.93)	0.62 (0.39–0.98)	0.73 (0.43–1.24)	3.19 (1.73–5.93)	1.21 (0.66–2.26)
≥ 60,000	1	1	1	1	1
Hospital					
Junior	0.13 (0.04–0.42)	0.05 (0.01–0.37)	0 (0–0)	4.33 (2.03–9.25)	0 (0–0)
Middle	0.74 (0.37–1.46)	0.15 (0.03–0.62)	0.18 (0.04–0.78)	5.13 (2.47–10.63)	0.44 (0.13–1.53)
Senior	1	1	1	1	1

*Y* Years, *ASM* Anti-seizure medication, *CI* Confidence interval, *OR* Odds ratio

^a^ Separate odds ratios adjusted for all of the variables in the model

## Discussion

In our study, we found that patients with a higher education level, aged <16 years, and with a higher personal income level were more likely to receive treatment from a neurologist. Patients with a lower personal income level who were treated at a junior hospital were more likely to receive prescriptions of carbamazepine, and those who were younger than 16 years were less likely to receive prescriptions of carbamazepine and oxcarbazepine. Patients with a higher education level, a higher income level, who were younger than 16 years and were treated at a senior hospital were more likely to receive prescriptions of levetiracetam. Adult, female patients with focal epilepsy treated at a senior hospital were more likely to receive prescriptions of lamotrigine.

Marked differences in the therapy methods were found in relation to age, educational level, place of residence, and personal income level. Patients who were young, more educated, from families with higher household income levels, and from a urban community were more likely to receive their prescriptions from a neurologist, which was consistent with the conclusion reported in a Swedish study [14]. Here, it should be noted that patients under 16 years were more likely than the elderly (> 60 years) to receive a prescription from a neurologist. A partial explanation for this finding might be due to the Chinese birth control policy (www.nhfpc.gov.cn/) that made the child, especially if they were older, more important than other members of the family, resulting in preferential behaviour and thus, an increased likelihood of treatment from a neurologist [15]. Rural residency had an influence on the ASMs prescribed by neurologists. This might be because of the geographic distribution of neurologists and the uneven number of the epileptic centers in western China. However, for patients from rural areas, the long travel distance to see a neurologist at a senior hospital might have been an obstacle to treatment. For example, it would take patients from the suburb of Chengdu more than 2 h to travel to our epilepsy center in the center of Chengdu; these results are similar to the results reported in Sweden [14]. Moreover, patients with higher educational levels and higher income levels were more likely to receive their prescription from a neurologist rather than from a non-neurologist specialist, which was also reported in the USA [16]. The reason for this phenomenon might be that patients with higher educational levels and higher income levels are willing to travel more and to try more treatments [17].

Patients with a lower personal income level who were treated at a junior hospital were more likely to receive prescriptions of carbamazepine. This medicine is commonly used because it is suitable for most epileptic seizure types [18]. Compared with elderly patients, younger patients were less likely to receive prescriptions of carbamazepine and oxcarbazepine. The reason for this phenomenon might be because oxcarbazepine, a new ASM, has some adverse effects such as fatigue, drowsiness, diplopia, dizziness, nausea, vomiting, and the common rash; thus, young patients may not be willing to choose it [19], which was also reported in a German study [17].

In this study, we found that patients with a higher education level, a higher income level, who were younger than 16 years and who were treated at a senior hospital were more likely to receive prescriptions of levetiracetam, which was also reported by Edward Faught [20]. This might be because of the good effectiveness and low risk of adverse drug reactions for levetiracetam [21]. Although this medicine might be more expensive compared with older drugs, it has some advantages, such as its efficacy in the treatment of focal epilepsy and its low rate of drug interactions, when compared with other ASMs [22]. Moreover, it can reduce epilepsy-related hospital encounters, leading to better patient adherence to therapy by indirect routes [23]. Adult, female patients with focal epilepsy treated at a senior hospital were more likely to receive a prescription for lamotrigine. Studies from Denmark, Norway and the UK also reported that women were more likely to receive lamotrigine [24]. The higher likelihood of receiving lamotrigine might be due to the following. 1) Lamotrigine monotherapy during pregnancy does not increase the risk of birth defects and other diseases, including autism spectrum disorder and attention-deficit/hyperactivity disorder [25, 26], though it is difficult to adjust the dose during pregnancy. 2) Lamotrigine monotherapy has few effects on the reproductive endocrine system [27, 28]. Due to better seizure control, valproate is still widely prescribed as a first-line treatment in patients with focal epilepsy, while valproate is rarely prescribed due to its intolerable adverse effects. This may be the reason that patients who were treated at a junior epileptic center were less likely receive valproate.

Our study has several limitations. First, the study did not record other characteristics of the patients or the epilepsy syndromes, such as adverse reactions, duration of epilepsy, or treatment success, which may have also influenced treatment choices. Second, these data on outpatients were from a single center, i.e., our epilepsy center at Sichuan Provincial People’s Hospital. Third, there may be a big difference in diagnosis between non-neurologists, such as between neurosurgery endocrinology, we neglect this difference in our study. At last, although this is a large size tertiary comprehensive hospital in western China and the patients were from different regions of western China, the sample size is relatively small. Future studies including multi-center and larger sample size studies are needed.

## Conclusion

Our study showed that sociodemographic characteristics are important for access to neurologists and the prescription of individual antiepileptic drugs. These data may help us to gain a better understanding of treatment disparities in western China and help public health officials to spread the guidelines among doctors and to create programmes to help patients according to their needs and the popularity of certain healthcare plans. Prospective studies are needed to assess whether the observed sociodemographic differences are important to patient-related outcomes such as seizure freedom and quality of life.

## Data Availability

All data generated or analyzed during this study are included in this published article. Further data set could be obtained on request if required. Our data are deposited in our epilepsy center database.

## Abbreviations

ASM: Anti-seizure medications
ILAE: International league against epilepsy
OR: Odds ratio
CI: Confidence interval

## References

[1] Singh A, Trevick S (2016). The epidemiology of global epilepsy. Neurol Clin.

[2] Gu L, Liang B, Chen Q, Long J, Xie J, Wu G (2013). Prevalence of epilepsy in the People's Republic of China: a systematic review. Epilepsy Res.

[3] Beghi E (2016). Addressing the burden of epilepsy: many unmet needs. Pharmacol Res.

[4] GBD 2016 Epilepsy Collaborators. Global, regional, and national burden of epilepsy,1990-2016: a systematic analysis for the Global Burden of Disease Study 2016. Lancet Neurol. 2019;18(4):357–75. 10.1016/S1474-4422(18)30454-X PMC6416168 30773428

[5] Patsalos PN, Spencer EP, Berry DJ (2018). Therapeutic drug monitoring of antiepileptic drugs in epilepsy: a 2018 update. Ther Drug Monit.

[6] Kwan P, Brodie MJ (2001). Effectiveness of first antiepileptic drug. Epilepsia.

[7] Brodie MJ, Barry SJ, Bamagous GA, Norrie JD, Kwan P (2012). Patterns of treatment response in newly diagnosed epilepsy. Neurology..

[8] Perucca P, Hesdorffer DC, Gilliam FG (2011). Response to first antiepileptic drug trial predicts health outcome in epilepsy. Epilepsia..

[9] Glauser T, Ben-Menachem E, Bourgeois B, Cnaan A, Guerreiro C, Kalviainen R (2013). Updated ILAE evidence review of antiepileptic drug efficacy and effectiveness as initial monotherapy for epileptic seizures and syndromes. Epilepsia..

[10] Glauser T, Ben-Menachem E, Bourgeois B, Cnaan A, Chadwick D, Guerreiro C (2006). ILAE treatment guidelines: evidence-based analysis of antiepileptic drug efficacy and effectiveness as initial monotherapy for epileptic seizures and syndromes. Epilepsia..

[11] Liu J, Liu Z, Chen T, Xu R (2013). Treatment of epilepsy in China: formal or informal. Neural Regen Res.

[12] Yu Z, Dong K, Chang H, Huang X, Ren Y, Fan C (2017). The epidemiological and clinical characteristics study on epilepsy in 8 ethnic groups of China. Epilepsy Res.

[13] Ding X, Zheng Y, Guo Y, Shen C, Wang S, Chen F (2018). Active epilepsy prevalence, the treatment gap, and treatment gap risk profile in eastern China: a population-based study. Epilepsy Behavior Epilepsy Behav.

[14] Mattsson P, Tomson T, Eriksson O, Brannstrom L, Weitoft GR (2010). Sociodemographic differences in antiepileptic drug prescriptions to adult epilepsy patients. Neurology..

[15] Xueyi Wang CZ (2013). Population age structure and consumption of health care in China. Statistical Study.

[16] Chen SY, Wu N, Boulanger L, Sacco P (2013). Antiepileptic drug treatment patterns and economic burden of commercially-insured patients with refractory epilepsy with partial onset seizures in the United States. J Med Econ.

[17] Hamer HM, Kostev K (2014). Sociodemographic disparities in administration of antiepileptic drugs to adults with epilepsy in Germany: a retrospective, database study of drug prescriptions. CNS Drugs.

[18] Nolan SJ, Marson AG, Weston J, Tudur Smith C (2015). Carbamazepine versus phenytoin monotherapy for epilepsy: an individual participant data review. Cochrane Database Syst Rev.

[19] Fang S, Gong ZC (2015). Adverse effects of oxcarbazepine. Zhongguo Dang Dai Er Ke Za Zhi.

[20] Faught E, Helmers SL, Begley CE, Thurman DJ, Dilley C, Clark C (2015). Newer antiepileptic drug use and other factors decreasing hospital encounters. Epilepsy Behav.

[21] Neininger MP, Ullmann M, Dahse AJ, Syrbe S, Bernhard MK, Frontini R (2015). Use of Levetiracetam in neonates in clinical practice: a retrospective study at a German University hospital. Neuropediatrics..

[22] Pickrell WO, Lacey AS, Thomas RH, Lyons RA, Smith PE, Rees MI (2014). Trends in the first antiepileptic drug prescribed for epilepsy between 2000 and 2010. Seizure..

[23] Beghi E (2012). Randomised controlled monotherapy trials: which comparators to use?. Epileptic Disord.

[24] Charlton R, Garne E, Wang H, Klungsoyr K, Jordan S, Neville A (2015). Antiepileptic drug prescribing before, during and after pregnancy: a study in seven European regions. Pharmacoepidemiol Drug Saf.

[25] Pariente G, Leibson T, Shulman T, Adams-Webber T, Barzilay E, Nulman I (2017). Pregnancy outcomes following in utero exposure to lamotrigine: a systematic review and meta-analysis. CNS drugs.

[26] Wiggs KK, Rickert ME (2020). Antiseizure medication use during pregnancy and risk of ASD and ADHD in children. Neurology..

[27] Sidhu HS, Srinivasa R, Sadhotra A (2018). Evaluate the effects of antiepileptic drugs on reproductive endocrine system in newly diagnosed female epileptic patients receiving either valproate or lamotrigine monotherapy: a prospective study. Epilepsy Res.

[28] Rauchenzauner M, Deichmann S, Pittschieler S, Bergmann M, Prieschl M, Unterberger I (2020). Bidirectional interaction between oral contraception and lamotrigine in women with epilepsy - role of progestins. Seizure..

